# Is Midlife Occupational Physical Activity Related to Disability in Old Age? The SNAC-Kungsholmen Study

**DOI:** 10.1371/journal.pone.0070471

**Published:** 2013-07-30

**Authors:** Elisabeth Rydwik, Anna-Karin Welmer, Sara Angleman, Laura Fratiglioni, Hui-Xin Wang

**Affiliations:** 1 Aging Research Center, Department of Neurobiology, Care Sciences and Society, Karolinska Institutet and Stockholm University, Stockholm, Sweden; 2 Research and Development Unit, Jakobsbergs Hospital, Stockholm County Council, Järfälla, Sweden; 3 Karolinska University Hospital, Stockholm, Sweden; 4 Stockholm Gerontology Research Center, Stockholm, Sweden; University of Valencia, Spain

## Abstract

**Objectives:**

Leisure-time physical activity (PA) has been established to be related to more years lived without disability. However, less is known about the relationship between occupational PA and disability in old age. The aim of the study was 1) to investigate whether midlife occupational PA is related to late-life disability, and 2) to test the hypothesis that the association differs according to the occupational categories of blue and white collar work.

**Methods:**

The study population was derived from the Swedish National Study on Aging and Care, and consisted of a random sample of 1804 subjects aged 72 and above. The association of occupational PA during the longest held occupation with disability in old age was determined using logistic regression.

**Results:**

There was no significant relationship between occupational PA and disability in personal or instrumental activities of daily living (ADL) after controlling for demographic and health-related factors. However, in stratified analyses moderate levels of occupational PA was associated with a lower odds ratio of dependency in personal ADL amongst white collar workers, compared to low level of occupational PA (OR = 0.34 95% C1 0.12–0.98).

**Conclusions:**

Moderate levels of midlife occupational PA were associated with a decreased risk of personal ADL disability in old age among white collar workers, but not among blue collar workers. Our results highlight the importance of encouraging white collar workers to engage in physical activity during or outside work hours.

## Introduction

Accumulated evidence has shown that leisure-time physical activity (PA) has a beneficial effect on various health outcomes. Specifically, non-occupational PA has been related to more years lived without disability, and higher levels of non-occupational PA has been shown to further increase the number of disability-free years and mortality [1], [2]. However, studies concerning occupational PA are limited and have shown contradictory results. Both low levels of leisure-time and occupational PA in middle-aged adults have been associated with excessive risk of ischemic heart disease [3]. Moderate to high levels of occupational PA have been suggested to protect against hip fracture [4], heart failure [5], myocardial infarction [6], and mortality [7]–[10], although the protective effect of occupational PA was weaker compared to leisure-time PA [4]–[5], [10]. Yet in another study occupational PA was not associated with future disability among middle-aged adults with self-reported arthritis over a 2-year follow-up [11]. On the other hand, high occupational PA has been related to an increased risk of cardiovascular mortality in middle-aged men with low and moderate aerobic fitness, but not among men with high level of aerobic fitness [12].

To date, population-based evidence on the relationship between midlife occupational PA and disability in old age is scarce. Strenuous occupational PA in competitive or recreational middle-aged runners has been associated with a greater likelihood of disability in their sixties [13]. In addition, it is known that blue collar workers are more likely to have a higher total PA level [14] and are at higher risk of developing arthritis or receiving a disability pension [15]–[17] and of developing disability and mobility limitation in old age compared to white collar workers [18]–[20]. Therefore, the association between occupational PA and late-life disability may differ among blue collar workers and white collar workers. To our knowledge, no studies have examined occupational PA in white collar workers in relation to disability in old age.

Given this evidence, we hypothesize that the association between occupational PA and disability in old age differs for white and blue collar workers. This study aimed: 1) to investigate whether midlife occupational PA is related to disability in old age, and 2) to test the hypothesis that the association between occupational PA and late-life disability differ according to the occupational categories of blue and white collar work.

## Methods

### Study Population

The Swedish National Study on Aging and Care in Kungsholmen (SNAC-K) is an ongoing longitudinal study in [21] Kungsholmen, a geographically-defined urban neighborhood of Stockholm, Sweden. The SNAC-K study population consists of a random sample of people within specific age cohorts, as described below, living either at home or in institutions. The baseline data collection was conducted during March 2001–August 2004. Eleven age cohorts were chosen with different intervals, with six years between groups in the younger cohorts (60, 66, 72 and 78 years old), and three years in the older cohorts (81, 84, 87, 90, 93, 96, 99 and above). The study population in SNAC-K has been enlarged by doubling the younger cohorts (aged 60–78 years), and an over-sampling of the 90+ population, due to the specific interest in longitudinal evaluation of the health and needs of care in the oldest-old. A total of 5111 persons were selected to be invited for participation. Of these 4590 were alive and eligible to participate (200 dead, 262 not able to be contacted, 4 deaf, 23 did not speak Swedish, 32 had moved), and of these 3363 (73.3%) participated at the baseline examination (1227 refused). The age groups 60 and 66 (n = 1304) were excluded from this analysis because they were either currently working or had only recently stopped working. Of the remaining 2059 participants, there were missing data for 248 participants regarding occupational PA and for two participants on instrumental activities of daily living (ADL), leaving 1809 participants in the analyses. There were no significant differences between the participants that had missing data and those included in the analyses in terms of personal and instrumental ADL, age, leisure-time PA, education, and socioeconomic position. However, participants with missing data were significantly more likely to be female, and to have multimorbidity (defined as two or more chronic diseases) and a lower cognitive level.

The study was approved by the Regional Ethical Review Board in Stockholm, Sweden. Informed consent in writing was obtained from all participants or their next of kin.

### Data Collection

The baseline data were collected through interviews, clinical examinations, testing and self-administered questionnaires performed by nurses, medical doctors and psychologists. For those who agreed to participate but were unable to come to the research center, home visits were conducted.

#### Disability

The subjects were asked if they independently managed both personal activities of daily living (PADL; dressing, hygiene, bathing/showering) and instrumental activities of daily living (IADL; cooking, cleaning, and running errands). If they answered no to any of these questions, they were coded as dependent in PADL or IADL, respectively. All subjects that lived in institutions were coded as dependent in IADL. If the subjects couldn’t provide information, a proxy, usually a next-of-kin was asked.

#### Occupational physical activity

The respondents were asked about the five longest previously held occupations. The subjects rated the PA level for each occupation according to seven levels from very light to very strenuous/full time. The occupational PA level was categorized into three groups in the later analyses: light (very light and light); moderate; and strenuous (strenuous and very strenuous some hours a day or full time). The occupational PA level during the longest held occupation was used in the analyses. If the subjects could not give information about time periods but gave information on type of work and PA level, the most recent work before retirement was used in the analyses.

#### Demographic data

Data regarding age, gender and highest level of formal educational level were recorded during the nurse interview. Socioeconomic position was derived from the longest held occupation and were categorized into three groups (according to classifications determined by Statistics, Sweden) [22]: 1) blue collar workers: no trained skill, goods-producing; no trained skill, service-producing; trained skill, goods-producing; trained skill, service-producing; 2) white collar workers: junior office worker, less than 2 years education after elementary school; junior office worker, ≥2 - <3 years after elementary school; office worker, ≥3 - <6 years after elementary school; senior office worker, ≥6 years after elementary school; 3) entrepreneurs: entrepreneurs; academic professions; farm owners.

#### Health-related factors

Chronic diseases were recorded by a study physician. To avoid misreporting and recall biases, different sources of medical diagnoses were used to limit potential errors. The classification was based on clinical examination, medical history, laboratory data and current drug use. A disease/condition was classified as *chronic* if it was prolonged in duration, and if one or more of the following characteristics were present: 1) leaving residual disability or worsening of quality of life; and/or 2) requiring a long period of care or treatment/rehabilitation. Number of co-occurring chronic diseases/conditions was recorded. Multimorbidity was defined as having ≥2 chronic diseases [23]. Cognitive level was measured with Mini Mental State Examination (MMSE) [24].

#### Leisure-time physical activity

Information on leisure-time PA was obtained through a self-administered questionnaire concerning both intensity and frequency. Intensity was divided into two levels: light exercises (walks on the sidewalk or paved surfaces, in parks, in forests, short bike rides, light aerobic or gym classes, golf) and moderate to intense exercises (brisk walking, jogging, heavy gardening, long bike rides, intense aerobic or gym classes, skating, skiing, swimming, ball games or similar activities). Information on frequency included: every day; several times a week; two-to-three times per month; less than two-to-three times per month; never. Following the recommendations by the World Health Organization and the American College of Sports Medicine [25]–[26], the participants were categorized into three different groups according to the intensity and frequency of the activities (from low to high): 1) inadequate: never, <2–3 times per month, 2–3 times per month in light and/or moderate/intense exercise; 2) health-enhancing: light exercise several times per week or every day; and 3) fitness-enhancing: moderate/intense exercise several times per week or every day [27].

### Statistical Analyses

Descriptive analysis was performed by presenting the number and the proportion of participants with light, moderate and strenuous occupational PA as well as dependency in PADL and IADL according to characteristics of the participants, and statistical differences were calculated using chi square tests.

Multinomial logistic regression analyses were conducted using dependency in personal and instrumental ADL, respectively, as the dependent variable and occupational PA level as the independent variable with having a light level of occupational PA level as the reference group.

Missing data for light (n = 707) and moderate to intense (n = 882) leisure-time PA were imputed in two steps. First, a manual imputation was performed. Subjects who were wheelchair bound (n = 37), could not move indoors or outdoors (n = 101), and who had severe physical problems or were incapable of walking 50 meters (n = 60) were coded as never taking part in any exercise. Secondly, we used fully conditional multiple regression imputation, which is a multivariate technique for multiply imputing missing values using a sequence of regression models [28]. The following variables were included in the imputation according to the literature: age; gender; light exercise; moderate to intense exercise; living in own home or institution; PADL; IADL; walking speed; number of chronic diseases; presence of dementia; chronic stroke; Parkinson’s disease; pain while moving; presence of emotional support; social anchorage; and locus of control. The imputation was repeated five times and the results were saved in five different data sets. The data sets were pooled into one dataset with five observations for each person and the mean of the five observations for each person were used in the analyses.

In the first set of models (model A) demographic factors were controlled for. The factors included were age, gender, education (below/above high school level), socioeconomic position, and number of years in the longest held occupation. In the second set of models (model B) the analyses were additionally adjusted for current health-related factors: multimorbidity (below/above ≥2 diseases), cognitive function, leisure-time PA as well as instrumental ADL disability in the personal ADL-analyses. To test the hypothesis that the associations between occupational PA and late-life disability are different according to the category of occupation, stratified analyses by blue collar workers and white collar workers (including entrepreneurs) were performed.

Because the youngest and the oldest age groups were oversampled, a weighted variable was created and used in all proportion (percentage) and aggregated analyses. The analyses were conducted in SPSS (Statistics 18.0) and Stata/SE 11.2.

## Results

Participants that were dependent in personal and instrumental ADL were more likely to be older, female, have lower education, multimorbidity, and lower cognitive function (Table 1). Participants who reported light occupational PA were more likely to be female, have higher education, white collar occupation and higher cognitive function (Table 2). Subjects with moderate or heavy occupational PA were more likely to have lower education and a blue collar occupation.

**Table 1:** Characteristics of the study population in relation to activities of daily living (ADL), n (%).

		Personal ADL		Instrumental ADL	
	Totaln = 1809	Dependentn = 189	Independentn = 1620	Dependentn = 682	Independentn = 1127
**Occupational physical activity**					
Light	1306	126 (66)	1180 (73)	478 (71)	828 (73)
Moderate	387	49 (26)	338 (21)	167 (23)	220 (20)
Strenuous	116	14 (9)	102 (6)	37 (6)	79 (7)
**Age groups (years)** *					
72–78	882	26 (14)	856 (53)	165 (24)	717 (64)
81–84	420	31 (16)	389 (24)	155 (23)	266 (24)
87–100+	507	132 (70)	375 (23)	363 (53)	144 (13)
**Female gender** *	1232	147 (75)	1088 (67)	494 (71)	738 (66)
**Education** * **, more than12 years**	368	18 (12)	350 (22)	96 (15)	272 (24)
**Socioeconomic status^¤#^**					
Blue collar	517	81 (40)	436 (27)	226 (31)	291 (26)
White collar	1142	101 (56)	1041 (64)	407 (62)	735 (66)
Entrepreneurs	148	6 (4)	142 (9)	48 (7)	100 (9)
**Multi-morbidity** * # **,** ≥**2 diseases**	1132	162 (85)	970 (60)	534 (22)	598 (78)
**Cognitive function (MMSE)** * #					
0–23	197	89 (44)	108 (6)	177 (23)	20 (2)
24–27	363	57 (29)	306 (19)	191 (28)	172 (15)
28–30	1240	43 (27)	1197 (75)	308 (49)	932 (83)
**Length of longest occupation,^¤^** m (sd)	1663	19.1 (16.9)	24.6 (12.5)	24.9 (11.6)	22.6 (15.2)
**Leisure time PA** *					
Inadequate exercise	711	168 (87)	543 (33)	464 (66)	247 (22)
Health-enhancing exercise	843	17 (10)	826 (52)	187 (28)	656 (60)
Fitness-enhancing exercise	255	4 (3)	251 (15)	31 (6)	224 (18)

n = number of subjects, m = mean, sd = standard deviation, MMSE = Mini Mental State Examination, PA = physical activity.

* p<0.05 for both personal and instrumental ADL, ¤ p<0.05 for instrumental ADL.

# There were missing data for socioeconomic status (n = 1), multi-morbidity (n = 5), MMSE (n = 9).

All proportions are weighted and the weighted variable is included in the chi square analyses.

**Table 2:** Characteristics of the study population in relation to occupational physical activity, n (%).

		Occupational physical activity				
	Totaln = 1809	Lightn = 1306	Moderaten = 387	Strenuousn = 116		
**Age groups (years)** *						
72–78	882	664 (51)	160 (41)	58 (50)		
81–84	420	298 (23)	92 (24)	30 (26)		
87–90+	507	344 (26)	135 (35)	28 (24)		
**Female gender** *	1232	889 (68)	291 (74)	52 (48)		
**Education** * **, more than 12 years**	368	320 (25)	43 (11)	5 (5)		
**Socioeconomic status** * #						
Blue collar	517	198 (14)	239 (62)	80 (68)		
White collar	1142	996 (77)	121 (32)	25 (24)		
Entrepreneurs	148	111 (9)	26 (6)	11 (8)		
**Dependency in personal ADL**	189	126 (8)	49 (10)	14 (11)		
**Dependency in instrumental ADL**	682	478 (34)	167 (38)	37 (32)		
**Multi-morbidity** # **,** ≥**2 diseases**	1132	809 (62)	250 (64)	73 (61)		
**Cognitive function (MMSE)** * #						
0–23	197	122 (8)	58 (13)	17 (14)		
24–27	363	230 (17)	100 (24)	33 (30)		
28–30	1240	947 (74)	228 (63)	65 (56)		
**Length of longest occupation** * **,** m (sd)	1661	24.1 (13.0)	22.8 (13.2)	27.0 (13.4)		
**Leisure time PA**						
Inadequate exercise	711	492 (36)	177 (46)	42 (35)		
Health-enhancing exercise	843	625 (50)	163 (42)	55 (49)		
Fitness-enhancing exercise	255	189 (14)	47 (12)	19 (16)		

n = number of subjects, ADL = Activities in Daily Living, m = mean, sd = standard deviation, MMSE = Mini Mental State Examination, PA = physical activity.

* p<0.01, ¤p<0.05.

# There were missing data for socioeconomic status (n = 1), Multi-morbidity (n = 5), MMSE (n = 9).

All proportions are weighted and the weighted variable is included in the chi square analyses.

Occupational PA was not significantly associated with either PADL disability (moderate occupational PA OR = 1.37; 95% CI 0.92–2.06 and strenuous occupational PA OR = 1.49; 95% CI 0.77–2.92), or IADL disability (moderate occupational PA OR = 1.18 95%; CI 0.91–1.54 and strenuous occupational PA OR = 0.88; 95% CI 0.56–1.39), as compared to light occupational PA in the unadjusted analyses. Although moderate occupational PA was related to a decreased risk and heavy PA was related to an increased risk of PADL, these associations were not statistically significant after controlling for demographic and health-related factors (Table 3).

**Table 3:** Odds ratios (95% confidence interval) of ADL disability in relation to occupational physical activity, from multi-nomial logistical regression analyses.

	Occupational physical activity		
	Light (n = 1306)	Moderate (n = 387)	Strenuous (n = 116)
**Personal ADL**			
Model A	1.0	0.87 (0.51–1.47)	1.79 (0.73–4.36)
Model B	1.0	0.84 (0.47–1.50)	1.73 (0.67–4.42)
**Instrumental ADL**			
Model A	1.0	1.06 (0.75–1.51)	0.92 (0.52–1.63)
Model B	1.0	0.98 (0.67–1.45)	0.74 (0.42–1.29)

Independency in personal ADL or instrumental ADL is the reference category, respectively.

Model A: Adjusted for age, gender, education, socioeconomic status, number of years in longest held occupation.

Model B: Based on model A with additional adjustment for multi-morbidity, cognitive function, leisure physical exercise and instrumental ADL in the personal ADL analyses.

**Figure 1:**
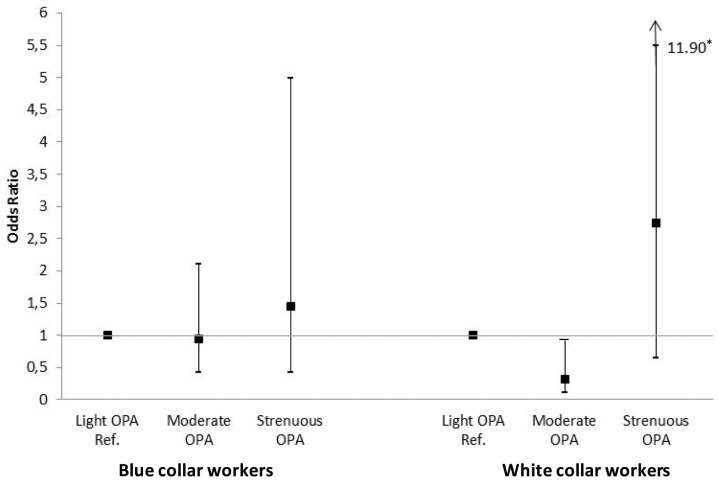
Odds ratios and 95% confidence interval of PADL disability in relation to occupational physical activity stratified by occupation, from multinomial logistic regression models, with light occupational physical activity (OPA) as reference, controlling for demographic and health-related factors. *The upper limit for the confidence interval for white collar/strenuous OPA is 11.90 as designated in the figure.

When stratifying for blue and white collar workers, moderate occupational PA was associated with a significantly decreased risk of dependency in PADL (OR = 0.29 95% C1 0.09–0.92) in white collar workers (Figure 1) but not in blue collar workers after controlling for demographic and health-related factors. Strenuous occupational PA was associated with a non-significant elevated risk of dependency in PADL risk for both blue and white collar workers. Occupational PA was not significantly related to IADL in either group (data not shown).

## Discussion

To our knowledge, this is the first study that has investigated the association between occupational PA during the longest held occupation and disability in old age and at the same time examined the associations among blue and white collar workers, respectively. The study showed that occupational PA in midlife was not associated with PADL or IADL disability in old age. However, midlife moderate occupational PA decreased the risk of late-life PADL disability among white collar workers. The most common occupations among the white collar workers with moderate occupational PA were in the areas of health care, sales, teaching and engineering.

The non-significant relationship between occupational PA and disability is in line with a previous study in middle-aged people with arthritis [11], and another study showing a significantly increased risk of disability with strenuous occupational PA [13], although we observed only a non-significant risk effect of strenuous occupational PA on PADL disability. The non-significant result is reasonable because it is known that levels of occupational PA are related to occupation itself. Levels of occupational PA are usually described in terms of physical load or physical risk factors such as bending, twisting, pushing, pulling and lifting in a repetitive manner [29]. The majority of the blue collar workers that reported strenuous occupational PA in this study were mechanics, construction workers and plumbers. High levels of occupational PA that are mostly found among types of work such as construction, agriculture, mining and production [30] have been shown to increase the risk of musculoskeletal disorders and of absence from work due to long-term sickness [15], [17], [31]. Similar to our study, low levels of occupational PA are common among types of work such as administration, management and programming [30], and they are related to increased time in sitting, which has been shown to increase the risk of all-cause mortality and obesity [32], [33]. Therefore, the beneficial effect of the occupational PA and the harmful effects of the occupation on disability might be cancelled out. Further, disability is a complex construct influenced by a variety of factors such as diseases, environment, demographic factors, psychosocial factors and physical fitness [34].

This is the first study showing that midlife moderate occupational PA in white collar workers was associated with a decreased risk of late-life disability. The result is in line with an earlier study showing that moderate to high occupational PA protected against hip fracture in women [4]. Similar with another study, we found that strenuous occupational PA was related to an elevated risk of disability, although not statistically significant, in both blue and white collar workers [35]. These results highlight the importance of encourage moderate but not strenuous occupational PA. The beneficial effect of moderate occupational PA in white collar workers should be emphasized because white collar workers might be more prone to long work hours which in turn have been shown to reduce leisure PA [14]. If white collar workers can be more physically active at work, the beneficial effect of occupational PA may be retained because both leisure-time and occupational PA have been associated with a reduced risk of diseases and mortality, although the effect of occupational PA was weaker than leisure-time PA [4]–[6], [10]. In addition, worksite exercise has been shown to improve physical fitness, work ability and sleep quality [36]–[38]. Our finding that moderate occupational PA reduced the risk of PADL disability even when health-related factors including leisure-time physical exercise was controlled for highlights the importance of encouraging and facilitating the possibility to perform PA during or in conjunction to work hours. Although, it has been shown that leisure-time exercise is of importance, occupational PA and household activities are also of importance in order to reach recommended levels [39].

In contrary to previous studies showing an increased risk of disability in relation to occupational PA [18], [19] in blue collar workers, we found that occupational PA did not relate to increased risk of late-life disability. Even though blue collar workers have a higher total PA [14] this may not always be beneficial because they are more subject to hazardous and/or repetitive working situations. Further, people in lower socioeconomic classes, measured with occupational category and/or education, have a higher risk of disability [31], [40] up to early old age [41]. The beneficial effect of occupational PA may not have enough power to counteract the negative effect of the occupational hazards. Therefore, it seems as though the quality of the PA is important.

A limitation of the study is that the study sample has a higher educational level compared to the Swedish average level, which hampers the generalization of the results. Further, data on leisure-time PA were collected only at late-life but not at midlife, which limits the possibility to examine the impact of midlife leisure activities on the studied association. However, we did stratified analysis for late-life leisure-time PA, similar results were observed among those who were doing inadequate exercise and those who were doing health-enhancing or fitness-enhancing exercise. This suggests that leisure PA might not moderate the studied association because those who are active in late-life were more likely to be active in midlife as well [42]. Another limitation is the retrospective design, since data on occupation and occupational PA was retrospectively collected, which may introduce recall bias. However, the recall bias could be of less importance because face-to-face interviews were conducted in all participants, and may lead to an underestimation of the studied associations. In addition, a representative random population-based sample of participants has been included and health measures such as multimorbidity and cognitive level have been objectively measured, which increases the validity of the study.

In conclusion, this study showed that midlife occupational PA was not associated with either PADL or IADL disability in old age. However, midlife moderate occupational PA was related to a decreased risk of late-life PADL disability among white collar workers, findings that need to be confirmed in further studies. The findings highlight the importance of socioeconomic factors related to PA-level and health related outcomes, such as disability. It also highlight the importance of encouraging and facilitating people performing PA during or outside work hours, especially for white collar workers who have less occupational related activities and taking into account both level and quality of PA.
